# A Plea for the Broader Treatment of Epilepsy1Read at the thirty-fourth annual meeting of the Medical Association of Central New York, held at Buffalo, N. Y., August 27 and 28, 1901.

**Published:** 1901-10

**Authors:** William P. Spratling

**Affiliations:** Sonyea, N. Y.; Medical superintendent, Craig Colony for Epileptics; member American Medico-Psychological Association, New York County Medical Society, Buffalo Academy of Medicine, Rochester Pathological Association; Secretary National Association for the Study of Epilepsy, etc.


### BUFFALO MEDICAL JOURNAL.

VOL. XLI.—LVII. OCTOBER, 1901. No. 3.

### ORIGINAL COMMUNICATIONS.

### A Plea for the Broader Treatment of Epilepsy.[1]

BY WILLIAM P. SPRATLING, M. D., Sonyea, N. Y.

Medical superintendent, Craig Colony for Epileptics; member American Medico-Psychological Association, New York County Medical Society, Buffalo Academy of Medicine, Rochester Pathological Association; Secretary National Association for the Study of Epilepsy, etc.

BECAUSE of the great range and variety of its symptoms, no one of which is pathognomonic, epilepsy is unlike any other disease, and because of its protean character, it is most difficult to treat. The old definition declaring it to be "a disease characterised by tonic and clonic convulsions and followed by a loss of consciousness;" is happily passing from the field of scientific medicine.

As difficult as it is to frame a satisfactory definition of insanity, it is more difficult still to formulate one that will describe epilepsy, for as yet its etiology is too obscure; its manifestations too varied and inconstant; its results too unequal; its pathology too little known. It is my conviction that a broader treatment of epilepsy cannot be successfully undertaken in the absence of a broader and clearer understanding of its many causes, forms, phases and manifestations; and I propose to speak of some of these at this time in the briefest manner possible. Like insanity, epilepsy is most likely to develop at certain ages, though its epochs are not so numerous as are those of insanity.

In a paper read by the writer before the New York County Medical Society in 1894, the data for which was obtained from a study of several thousand cases admitted during a period of fifteen years to the New Jersey State Hospital at Morristown,

1. Read at the thirty-fourth annual meeting of the Medical Association of Central New York, held at Buffalo, N. Y., August 27 and 28, 1901.

reference was made to six distinct epochs at which insanity was likely to occur, all of them being strictly physiological save the epoch of heredity, that being patho-physiological, the term being used to include several cases in which insanity developed in the offspring at the same age it appeared in the parent.

As yet, we have been unable to establish a corresponding epoch in epilepsy, though I would not say that it does not exist. Early childhood, from the sixth month to the seventh year; puberty, in the female, from the thirteenth to fifteenth year; in the male, from the fourteenth to the sixteenth year; maternity; the epoch of heredity; the menopause and senility, were the six epochs named.

The relative frequency with which epilepsy and insanity arise at the different epochs they share in common, differs greatly. Epilepsy is vastly more common in childhood than later in life, while insanity is rare in childhood. In fact, of the six epochs, they share but three in common: early childhood, when, as just stated, insanity is rare and epilepsy common; puberty, when either epilepsy or insanity may arise in equal proportions; and senility when insanity, or physiological decay is common and genuine epilepsy extremely rare.

In mentioning these coincident periods that favor the development of these two diseases, let me call your attention to the important fact, that in their effects on the mind they are much alike; both attacking the individual when the organism is passing through a period of profound physiological disturbance, and is least fitted to withstand it, both having a common basis in the brain, and both impairing to some degree, the faculties of the mind.

Epilepsy is essentially a disease of early life. The epileptic age begins in infancy and terminates in nearly 85 per cent of all cases before the twentieth year; and in 16 per cent. of all cases, it comes on before the end of the third year.

What causes its development so early in life? To this there is but one answer, and that is, heredity and stress. Under heredity, we have as factors in the family that predispose to, or cause the disease in the offspring in the order of their importance: epilepsy, alcoholism, insanity and tuberculosis, and in some cases to an extent not yet determined, syphilis, when it affects the central nervous system, and possibly rheumatism.

Under stress, we have the following causes acting intrinsically or extrinsically, and which do not require a soil prepared by heredity to cause the disease, but which makes its origin more certain if such preparation has been made: external violence—such as a blow on the head; internal violence—such as a hemorrhage in the brain, or on the brain; psychic—such as shock from any cause; chemical—such as the presence of toxemic substances in the gastro-intestinal canal.

First taking up epilepsy itself in the parent as a cause of the disease in the child, we found that out of a total of 1070 cases—660 males and 410 females—it was transmitted to 15 per cent. of the males and 17 per cent. of the females, or 16 per cent. all told. Alcoholism was established as a family cause in the same 1070 cases, in 111 males and 51 females; 16 per cent. of the former, and 12 per cent. of the latter, the aggregate for the two being 14 per cent. Insanity in the parents, in the same cases caused the disease in 49 males and 42 females; 7½ per cent. of the former and 10½ per cent. of the latter.

It seems clear that more males acquire epilepsy as a result of alcoholism in the parent than do females, while more females acquire it as a result of insanity in the parent than do males. Tuberculosis was definitely established in the immediate ancestors in 101 males or 12 per cent., and in fifty females or 14 per cent. the two combined giving over 13 per cent. Collectively, we found either epilepsy, alcoholism, insanity or tuberculosis, and in some instances two or more of all these, to be hereditary factors in 56 per cent. of the 1070 cases studied; leaving 44 per cent. not due to inherited causes.

Now besides the active and important part played by heredity in this disease, we must also consider the influence of stress and can best do it under the subdivision laid down above. As to the frequency with which external violence causes epilepsy, I can present no figures at this time, and only make the general statement that trauma is sometimes responsible for the disease. Children often get a blow on the head and nothing is thought of it until years after when convulsions arising, the blow is recalled, and the convulsions ascribed to it. We ought to be careful in accepting without challenge such a cause, especially in cases when heredity is bad.

A blow on the head that crushes the skull so that it injures brain tissue, and is followed by convulsions, does not to my mind any more imply the presence of epilepsy than do the convulsive movements of the frog subjected after decapitation to the irritation of a galvanic current.

Convulsions alone do not constitute epilepsy, but in a case of brain injury, unless the irritation is removed within a reasonable time, and the length of this time depends upon the stamina of the individual, the convulsions may become fixed, and eventually acquire all the characteristics of, and pass into true epilepsy. There is but one safe rule to follow in such cases, and that is to repair the injury as soon as possible.

Internal violence: hemorrhage within the brain or beneath its coverings, was found to have caused the disease in 116 cases or 11 per cent. of the entire number studied.

The brain palsies of early life that cause epilepsy may be manifested in the form of hemiplegia, paraplegia, or diplegia; the two latter being comparatively rare. The seat of the lesion in these cases is to be found in the hemispheres; the central motor neurons being involved in that part of the direct motor tract which extends from the brain cortex to the spinal cord as far as the anterior horn.

Brain palsies more often occur during the first three years of life. About one-third of all cases are congenital, the rest being due to injuries received at birth; to the stress of dentition in favorable subjects, and to causes that lead to embolism and thrombosis; these being injuries and the infectious fevers, such as pneumonia, whooping cough, measles and scarlet fever.

Sixty-seven of the 116 cases were males and 43 females—62 suffering from left and 51 from right hemiplegia; 3 being diplegics.

We have often seen epileptics whose disease was caused by cerebral palsy, and in which the cause had gone unrecognised because the marks of the paralysis had become so indistinct.

It often requires the most careful inspection with the patient completely stripped, with accurate comparative measurements of the legs and arms, the testing of the muscular power and of the various reflexes, in addition to a close and systematic inquiry into the patient's early personal and family history, to bring the true cause to light.

There is no question but what the stress of psychic violence, if I may so use these terms, sometimes causes epilepsy. Three cases of the kind in young children have come under my observation during the past two years. It was caused in two of them by the violence of a drunken father, and in the other by the attack of a vicious dog.

Chemical causes, in my opinion, play an important part in the production of epilepsy, and by chemical causes I mean all toxic agencies generated in the body or introduced from without.

It seems that there must be some local relationship between the gastric aura, which we find in nearly 20 per cent. of all cases, and the epilepsies due to gastro-intestinal poisoning; nor is it unreasonable to suppose that serum-therapy may not some day be used to advantage in the treatment of cases of this kind. This is not surprising after the statements recently made by Dr. Ford Robertson, pathologist to the Scottish asylums, that paresis is not primarily a disease of the brain, but has its origin in toxemic conditions in the intestinal canal.

So far we have learned two things: (1) that nearly 85 out of every 100 epileptics acquire the disease before they reach the age of 20 years; (2) that the four factors in heredity: epilepsy alcoholism, insanity and tuberculosis, in addition to the cerebral palsy cases of early life, constitute 67 per cent. of all the cases we have to deal with.

These facts furnish a good working basis for treatment thus far. It teaches us that the time to treat epilepsy is when the epileptic is young and the disease but recently developed. Not many cases of epilepsy can be cured, but no one can say off-hand what case is curable and what is not.

I believe that much of the surgical work done for the relief of epilepsy should not be done. In some cases the operation is put off until the disease has become so thoroughly established that it is not removable by any means, and least of all can it be excised with the knife. Again, I have seen many trephinings done in cases in which the epilepsy was unquestionably inherited. I believe the great principle of *non-nocere* should be made to apply in these cases. Before we do any sort of an operation for the relief of epilepsy, we ought to study the case closely from every side, taking into account hereditary factors, if any; the character, frequency and duration of the seizures, and especially should we consider the mental condition of the patient at the time; for if this has been impaired, I question whether an operation is ever justifiable as a means of cure; but I do not say it should not be done as a palliative measure.

Nothing is further from my mind than to create the impression that epilepsy is incurable and that but few, or none, can be benefited, for I know by experience that some of these cases can be cured, and that fully 75 per cent. of all—irrespective of cause or condition—under systematic and long-continued treatment can be greatly benefited. I only plead for a closer inquiry into the causes of the disease, in order that broader forms of treatment may be instituted.

DISCUSSION.

Dr. F. S. Crego, Buffalo.—In regard to Dr. Spratling's paper, I wish to say that my observation has directed me to believe that injury to the skull is a very much greater factor in the causation of epilepsy than the doctor has given it in his paper. I think a large number of these cases that are put down as hereditary are really due to injury to the skull during birth, during the passage of the head through the straits, and I have confirmed that by a large number of post-mortems on epileptic children; and in view of that fact I am a great believer in injury to the skull as a cause of epilepsy, and am not a great believer in inheritance. I think a large portion of cases of epilepsy arise from injury to the skull. The paper is very interesting indeed, and especially the paper of Dr. Clark is interesting in view of the fact that a large number of these cases are cured now since the new method of treatment, the salt starvation, has been introduced.

Dr. L. P. Clark, Sonyea.—I would say in regard to the liability to injury during birth, and that being a cause of epilepsy, it is held so frequently to be the case by a number of obstetricians that recently I had occasion to go over statistics of several thousand epileptics, and in each instance the cases were all carefully recorded. I found in that that it was ten to one, the prolonged labor against a possibility of trauma from obstetrical intervention. I think that one thing that general practitioners, and especially obstetricians, should bear in mind in regard to the birth of neurotic children, is, that the prolonged labor is ten times to one more liable to cause nervous lesion and neuroses and things of that sort than that of instrumental delivery. Prolonged labor and its attendant changes is to me one of the most frequently observed causes in the epileptic etiology, and I think Freud and Gowers, and Wollenberg, of Vienna, have recently again called attention to the fact that the dangers of prolonged labor are very great indeed, and that skilled attention from the hands of obstetricians should be given early, and not permit the child to suffer all the attendant evils which prolonged labor would induce.

Dr. Wm. C. Krauss, Buffalo.—I would like to ask Drs. Spratling and Clark what number of cases are being trephined for traumatic causes?

Dr. Spratling.—We have received a large number of epileptics who have been trephined. We have operated on a few ourselves, in which we have taken into consideration hereditary factors, but so far we have been unable to benefit a case permanently.

Dr. Krauss.—I had a case in 1891 which Dr. Mynter operated on for me, where the patient had received injury to the frontal bone by an explosion. There was a well-marked depression from below, and at the time of the operation the patient was in a condition of status epilepticus. Quite a large portion of the frontal bone was removed and the patient recovered, although he used the bromides for several months afterward. Sometime after the operation the attacks reappeared, almost as severe as before. He underwent a second operation. The attacks ceased for about two years. They reappeared, and a third operation was undertaken. Almost the whole of the frontal and part of the parietal bone were removed, and now the attacks are practically as severe as ever. I have at the present time two or three cases of epilepsy where the origin is supposed to be traumatic, and I have very hard work to keep the family from having an operation. I told them repeatedly that the chances are that an operation will do no good at all, and that the bromide treatment, or the modified treatment, and the hygienic treatment is about as good a treatment as can be given. I am very glad to hear from the two physicians from the Craig Colony, whose experience is perhaps the best in this country.

Dr. S. F. Snow, Syracuse.—I do not wish to be considered an epileptic specialist or expert, but a case of epilepsy came under my care about seven years ago that had quite a particular interest in it. The patient had been through a thorough course of treatment with bromide, and with the varying results we all know so well of, before the idea of salt starvation came in. She was sent to me, I think, by Dr. Young, of Newark. Following along the line of the thought that there must be some nerve irritation, I examined the interior of the nose and found a badly deflected septum, with a heavy outgrowth; and not knowing surely that this would help the patient, still on general principles I advised the removal of that outgrowing septum, and I removed that. There was a marked improvement in the case from the removal of that pressure, and this improvement continued as long as we could keep the nasal membrane in a fairly healthy condition. The patient, from having a seizure every two or three days, would sometimes go two months without them, and she became quite a useful member of society. I have heard that she was engaged as an organist. There was a time for two or three years that she remained better. Then the seizures returned, and she came to me and made arrangements for straightening the nasal septum. I wished to see her at the hospital, but when she got into our city she fell into the hands of a homeopath, who advised an operation of trephining through the skull, and I understand she went through it and died. That case taught me that there are a few of these people who have their seizures through pressure within the nose. I don't know what the percentage may be, but I have felt very much impressed with it.

Dr. L. A. Weigel, Rochester.—Dr. Clark referred to prolonged labor as a possible cause of epilepsy, and I would like to ask him whether the other conditions, of premature labor, and so on, might not have a similar influence. Some years ago Dr. Little, of London, wrote rather a classical paper on the nerve phenomena that follow those conditions, premature labor, prolonged labor, instrumental delivery and asphyxia, and in all of the cases of so-called spastic paralysis that I have seen, every case, if I remember rightly, has given the history of either one of those conditions. Dr. Little ascribed the damage to the brain, to capillary hemorrhage, which later on produced the destructive changes in the brain tissue.

Dr. Clark, Sonyea.—Replying to Dr. Weigel's inquiry, I will say that those experiences have been mine exactly, that the premature labor, prolonged labor and instrumental delivery are productive of the spastic cases of surface hemorrhages, and Bashaw, of London, who has written extensively on the matter, and Mr. Nutt, in this country, have demonstrated that the prolonged labor has been the cause that in the majority of cases has produced this type.

Dr. Spratling, Sonyea.—Dr. Crego, I am afraid, got the idea that I do not believe that trauma can cause epilepsy. I attempted to state clearly that I think it does sometimes cause it, but I made a distinction between epilepsy caused by injury in utero and cases of injuries received afterward. I class them all as among the palsy cases of early life.

